# Clinicopathological significance and exploratory prognostic modeling of USP18 and YBX3 expression in clear cell renal cell carcinoma: a retrospective Chinese cohort study

**DOI:** 10.3389/fonc.2026.1897469

**Published:** 2026-08-04

**Authors:** Qikai Zou, Siqi Chen, Yanping Li, Xin Chen, Zhijie You, Xin Chen, Chen Wang

**Affiliations:** 1 School of Basic Medical Sciences, Fujian Medical University, Fuzhou, China; 2 Department of Pathology, Shengli Clinical Medical College of Fujian Medical University, Fujian Provincial Hospital, Fuzhou University Affiliated Provincial Hospital, Fuzhou, China

**Keywords:** clear cell renal cell carcinoma, clinical prediction model, immunohistochemistry, prognosis, ROC curve, survival analysis, USP18, YBX3

## Abstract

**Background:**

Clear cell renal cell carcinoma (ccRCC) is clinically heterogeneous, and tissue biomarkers may complement conventional pathological risk assessment. Ubiquitin-specific protease 18 (USP18) and Y-box binding protein 3 (YBX3) have been implicated in tumor progression, but their clinicopathological and prognostic relevance in ccRCC remains uncertain.

**Methods:**

We retrospectively evaluated 98 patients with pathologically confirmed ccRCC using paired tumor-adjacent immunohistochemistry, clinicopathological correlation, Kaplan-Meier analysis, Cox regression, and exploratory receiver operating characteristic modeling. Immunohistochemical scores were analyzed continuously as the primary analytical scale; a cohort-median cutoff of ≥6 was used only for descriptive and exploratory survival stratification.

**Results:**

USP18 and YBX3 scores were higher in tumor than paired adjacent renal tissue (both p < 0.001) and were moderately correlated (Spearman rho = 0.540, p < 0.001). USP18-high expression was not associated with most clinicopathological variables and was not significant in categorical univariable survival analysis (HR 3.59, 95% CI 0.71-18.28; p = 0.124). YBX3-high expression was associated with inferior overall survival by the log-rank test (p = 0.047), while combined high expression showed exploratory survival separation (p = 0.019). Continuous USP18 and YBX3 scores were associated with mortality in univariable Cox models, but only 12 deaths occurred, precluding reliable multivariable biomarker validation or adequately powered interaction testing. Apparent in-sample AUC gains after adding biomarkers to clinical variables were small and unvalidated.

**Conclusion:**

USP18 and YBX3 are overexpressed and co-expressed in ccRCC. The evidence is stronger for YBX3 than for categorical USP18 expression, and the combined signal should be regarded as hypothesis-generating rather than proof of synergy or independent prognostic utility. Larger externally validated cohorts with prespecified cutoffs are required.

## 1 Introduction

Renal cell carcinoma (RCC) is a common malignant tumor of the urinary tract, and clear cell renal cell carcinoma (ccRCC) is its predominant histological subtype. ccRCC is clinically heterogeneous: some tumors remain organ-confined, whereas others develop recurrence, metastasis, or treatment resistance despite similar conventional pathological features (1, 2). This variability limits the ability of stage, grade, and tumor size alone to fully explain clinical outcomes after surgery.

Routine pathological assessment of ccRCC includes tumor stage, WHO/ISUP grade, tumor necrosis, tumor size, vascular invasion, capsular invasion, and evidence of regional or distant spread. These factors remain central to risk stratification because they reflect architectural, cytological, and invasive tumor behavior (3, 4). However, patients with similar clinicopathological profiles may still show divergent survival patterns. This has encouraged the study of tissue biomarkers that can be assessed in formalin-fixed, paraffin-embedded material and integrated with routine pathological variables.

The ubiquitin-proteasome system is an important regulator of protein stability, signaling, cell-cycle control, and cellular stress adaptation. Deubiquitinating enzymes can remove ubiquitin from substrate proteins, inhibit proteasomal degradation, and alter protein localization or function (5). In kidney cancer, deregulated deubiquitination has been linked to tumor-promoting pathways, including USP13-mediated stabilization of ZHX2 and USP37-mediated stabilization of HIF-2α (6, 7).

Under physiological conditions, ubiquitin-specific protease 18 (USP18) is an interferon-inducible, ISG15-specific cysteine protease that removes ISG15 from conjugated proteins and helps maintain cellular ISGylation homeostasis. Independently of its catalytic activity, USP18 also provides negative-feedback control of type I interferon signaling by limiting sustained signaling through the IFNAR2-JAK-STAT pathway (8–10). These complementary functions protect cells from excessive interferon-driven inflammation and stress. In cancer, however, dysregulated USP18 may be co-opted to support proliferation, apoptosis resistance, metabolic adaptation, and invasion (11–15). Its clinicopathological significance in ccRCC therefore requires evaluation in the context of both its normal immune-regulatory function and its potential tumor-associated activity.

Y-box binding protein 3 (YBX3), also known as CSDA or dbpA, is a ubiquitously expressed cold-shock-domain RNA-binding protein with physiological roles in post-transcriptional gene regulation. YBX3 binds selected messenger RNAs and can influence their stability and translation, including transcripts encoding solute carriers involved in amino-acid transport and cellular metabolic homeostasis (16). It also participates in cellular stress adaptation, proliferation, and differentiation (17). In malignant cells, disruption of these normal regulatory functions has been associated with enhanced migration, invasion, metastatic potential, and unfavorable tumor biology (18, 19). In ccRCC, YBX3 has been identified as an oncogenic RNA-binding protein associated with disease progression (20). This progression from physiological RNA regulation to tumor-associated dysregulation provides a biological rationale for evaluating YBX3 together with USP18 in human ccRCC tissue.

A clinically relevant question is whether USP18 and YBX3 provide reproducible prognostic information individually or whether their correlated expression identifies a distinct higher-risk phenotype. We therefore evaluated USP18 and YBX3 immunohistochemical expression in a retrospective ccRCC cohort, assessed their clinicopathological and survival associations, and explored whether adding either or both biomarkers changed the apparent discrimination of clinicopathological prediction models. Because the cohort contained few death events, all prognostic modeling was prespecified as exploratory rather than confirmatory.

## 2 Materials and methods

### 2.1 Study design and reporting approach

This retrospective tissue-based study integrated a clinicopathologically annotated ccRCC cohort, paired tumor-adjacent histological assessment, immunohistochemistry, survival analysis, and exploratory clinical prediction modeling. The study was designed to determine whether USP18 and YBX3 expression were associated with tumor behavior and whether these biomarkers provided apparent prognostic information beyond clinicopathological variables. Reporting followed the Reporting Recommendations for Tumor Marker Prognostic Studies (REMARK), where applicable (21).

### 2.2 Patient cohort, eligibility criteria, and tissue collection

The institutional cohort consisted of 98 patients who underwent radical nephrectomy, partial nephrectomy, or local ablation at Fujian Provincial Hospital between January 2014 and December 2019 and had pathologically confirmed ccRCC with adequate archived diagnostic material. Formalin-fixed, paraffin-embedded tumor tissue and paired adjacent non-neoplastic renal tissue were obtained from the institutional archive. Ablation cases were included only when adequate tumor and paired adjacent tissue were available for histological and immunohistochemical evaluation. The adjacent specimens were histologically non-neoplastic renal tissue obtained from the same patients and processed in parallel with the tumors. They were used as matched internal tissue comparators rather than as an independent healthy or population-based non-cancer control group.

Inclusion criteria were confirmation of ccRCC by at least two pathologists, availability of adequate tumor and paired adjacent tissue for histological and immunohistochemical analysis, and availability of clinicopathological and follow-up data sufficient for survival analysis. Exclusion criteria were non-clear-cell RCC, another simultaneous renal cancer, another tumor type, or absence of critical clinicopathological or follow-up information.

Demographic data, surgical approach, maximum tumor diameter, vascular invasion, lymph-node or distant-organ metastasis, Ki-67 index, pathological stage, WHO/ISUP grade, capsular invasion, intratumoral necrosis, recurrence or metastasis during follow-up, survival status, and follow-up duration were collected from pathology reports and medical records. Overall survival was calculated from surgery to death from any cause or final follow-up. Patients alive or lost to follow-up were censored at the date of last contact.

### 2.3 Histopathological review and immunohistochemical assessment

Hematoxylin and eosin-stained slides were reviewed to verify the diagnosis and select representative tumor and adjacent renal tissue sections. Tumors were classified according to the fifth edition of the WHO Classification of Tumours of the Urinary and Male Genital Systems (4). Nuclear grade was assigned using the WHO/ISUP grading system. Intratumoral necrosis, vascular invasion, capsular invasion, and lymph-node or distant-organ metastasis were recorded from histopathological and clinical documentation.

Immunohistochemistry was performed on 5-µm formalin-fixed, paraffin-embedded sections. Sections were deparaffinized in xylene, rehydrated through graded ethanol, and subjected to heat-induced epitope retrieval before endogenous peroxidase and non-specific binding were blocked. The primary antibodies were affinity-isolated rabbit polyclonal anti-USP18 (Prestige Antibodies^®^ powered by Atlas Antibodies, Sigma-Aldrich/Merck, St. Louis, MO, USA; catalog no. HPA044768; RRID: AB_10963697) and affinity-isolated rabbit polyclonal anti-YBX3/CSDA (Prestige Antibodies^®^ powered by Atlas Antibodies, Sigma-Aldrich/Merck; catalog/SKU no. HPA034838-100UL; lot no. 000047760; vial concentration 0.18 mg/mL; RRID: AB_10670980). Both products are specified by the manufacturer for human immunohistochemistry, with a recommended dilution range of 1:200-1:500. The exact working dilutions used in this retrospective series were not retained in the archived laboratory records and therefore cannot be reported with certainty. Bound primary antibody was detected using an HRP-conjugated anti-rabbit polymer system and visualized with 3,3′-diaminobenzidine, followed by Mayer-type hematoxylin counterstaining, dehydration, clearing, and mounting. The xylene, ethanol, antigen-retrieval, blocking, detection, chromogen, counterstain, dehydration/clearing, and mounting reagents were procured from Sigma-Aldrich/Merck. Tumor and paired adjacent sections were processed in parallel under the same conditions. Product-specific catalog and lot identifiers for the anti-USP18 vial and the ancillary Sigma-Aldrich reagents were not retained in the retrospective archive and therefore are not reported.

### 2.4 Immunohistochemical scoring and cutoff definition

Immunoreactivity was scored semi-quantitatively by combining staining intensity and the proportion of positive cells. Staining intensity was graded as 0 (negative), 1 (weak), 2 (moderate), or 3 (strong). The proportion of positive cells was graded as 0 (0%), 1 (1%-25%), 2 (26%-50%), 3 (51%-75%), or 4 (76%-100%). The final IHC score was calculated as intensity multiplied by extent, producing a score from 0 to 12.

The median IHC score was 6 for both USP18 and YBX3 in this cohort. Accordingly, a score ≥6 was used as a cohort-median threshold to provide descriptive high/low categories and clinically interpretable Kaplan-Meier plots. This threshold was not derived from an external validation cohort and should not be interpreted as a clinically validated decision cutoff. To reduce information loss and cutoff-dependent inference, continuous IHC scores were used as the primary biomarker variables in Cox regression and ROC-based modeling.

### 2.5 Statistical analysis and exploratory clinical prediction modeling

Continuous variables were summarized as mean ± standard deviation and median with interquartile range, as appropriate. Categorical variables were summarized as numbers and percentages. Tumor-versus-adjacent IHC comparisons were evaluated using paired t-tests and Wilcoxon signed-rank tests. Normality was assessed using the Shapiro-Wilk and Kolmogorov-Smirnov tests. Because IHC scores and several continuous variables were non-normally distributed, subgroup comparisons of continuous scores used Mann-Whitney U tests for two groups and Kruskal-Wallis tests for stage and grade categories.

Associations between biomarker high/low status and clinicopathological variables were evaluated using Pearson chi-square tests or Fisher exact tests when expected cell counts were small. The relationship between USP18 and YBX3 was evaluated using categorical association analysis, Spearman rank correlation, and Pearson correlation. Overall survival was analyzed using the Kaplan-Meier method and compared using the log-rank test (22). Univariable Cox proportional-hazards models estimated hazard ratios (HRs) and 95% confidence intervals (CIs) (23).

Only 12 deaths occurred. The principal survival analysis therefore emphasized univariable estimates, effect sizes, and confidence-interval precision. The previously constructed multivariable models are reported only as exploratory sensitivity analyses in **Supplementary Table 1** and were not used to claim independent prognostic validation. The number of candidate predictors and the wide confidence intervals indicate a substantial risk of overfitting.

A USP18×YBX3 interaction term was considered because the two continuous IHC scores were moderately correlated. It was not fitted in the primary model because the combination of only 12 death events and correlated predictors would make the interaction estimate highly unstable and vulnerable to multicollinearity and sparse-data bias. Consequently, the manuscript uses the terms co-expression and combined expression rather than statistical synergy. A definitive interaction analysis will require a substantially larger cohort and centered continuous biomarker scores.

ROC analysis evaluated apparent discrimination for death/event and metastasis/recurrence. Logistic regression models used clinicopathological variables alone and after adding continuous USP18, continuous YBX3, or both scores. Clinical variables included age, tumor size, grade group, stage group, capsular invasion, necrosis, vascular invasion, and lymph-node or distant-organ metastasis, where appropriate. LDH, serum calcium, and hemoglobin were available for only 65 patients and were not included in the main models. Because models were developed and evaluated in the same dataset without internal or external validation, AUCs are reported as apparent in-sample estimates and not as validated performance measures. No conclusion of incremental clinical utility was based on small AUC differences alone.

All tests were two-sided, and p < 0.05 was considered statistically significant. Given the exploratory design and multiple analyses, p-values were interpreted together with effect sizes, confidence intervals, consistency across analyses, and biological plausibility. Analyses were conducted using SPSS and complementary statistical software.

## 3 Results

### 3.1 Clinicopathological characteristics of the cohort

A total of 98 patients with pathologically confirmed ccRCC were included. The cohort included 62 males (63.3%) and 36 females (36.7%), with a mean age of 57.80 ± 12.30 years. Patients older than 55 years accounted for 54 cases (55.1%). Radical nephrectomy was performed in 61 cases (62.2%), and partial nephrectomy or ablation was performed in 37 cases (37.8%). The mean maximum tumor diameter was 4.37 ± 2.55 cm, and 14 tumors (14.3%) were larger than 7 cm. Stage I-II disease accounted for 89 cases (90.8%), while stage III-IV disease accounted for 9 cases (9.2%). Grade 3–4 tumors were present in 46 cases (46.9%). Vascular invasion, lymph-node or distant-organ metastasis, capsular invasion, and necrosis were present in 23 (23.5%), 15 (15.3%), 71 (72.4%), and 12 (12.2%) cases, respectively. Metastasis or recurrence during follow-up occurred in 21 patients (21.4%), and 12 patients (12.2%) died during follow-up (**Table 1**).

**Table 1:** Baseline demographic, pathological, treatment, and outcome characteristics of the retrospective ccRCC cohort (N = 98).

Variable	n/summary
Sex: male	62 (63.3)
Sex: female	36 (36.7)
Age >55 years	54 (55.1)
Age ≤55 years	44 (44.9)
Surgery: radical nephrectomy	61 (62.2)
Surgery: partial nephrectomy/ablation	37 (37.8)
Tumor size >7 cm	14 (14.3)
Tumor size ≤7 cm	84 (85.7)
Vascular invasion present	23 (23.5)
Lymph-node/distant-organ metastasis present	15 (15.3)
Stage I	77 (78.6)
Stage II	12 (12.2)
Stage III	9 (9.2)
Stage IV	0 (0.0)
Stage III-IV	9 (9.2)
Grade 1	3 (3.1)
Grade 2	49 (50.0)
Grade 3	42 (42.9)
Grade 4	4 (4.1)
Grade 3-4	46 (46.9)
Capsular invasion present	71 (72.4)
Necrosis present	12 (12.2)
Metastasis/recurrence during follow-up	21 (21.4)
Death during follow-up	12 (12.2)
Age, years	57.80 ± 12.30; median 57.50 (50.00-66.75); range 27.00-86.00
Maximum tumor diameter, cm	4.37 ± 2.55; median 4.00 (2.50-5.50); range 0.70-12.50
Ki-67 index	0.12 ± 0.21; median 0.08 (0.05-0.10); range 0.01-2.00
Survival/follow-up time, months	71.98 ± 35.22; median 76.00 (49.50-97.75); range 1.00-133.00

Values are n (%) unless otherwise indicated. Continuous variables are reported as mean ± standard deviation, median (interquartile range), and observed range. Tumor stage was assigned according to the applicable TNM/WHO framework, and nuclear grade was assigned using the WHO/ISUP system. Overall survival/follow-up time was measured from surgery to death from any cause or last recorded contact. ccRCC, clear cell renal cell carcinoma; SD, standard deviation; IQR, interquartile range.

### 3.2 Tumor-versus-adjacent immunohistochemical expression of USP18 and YBX3

**Figure 1:**
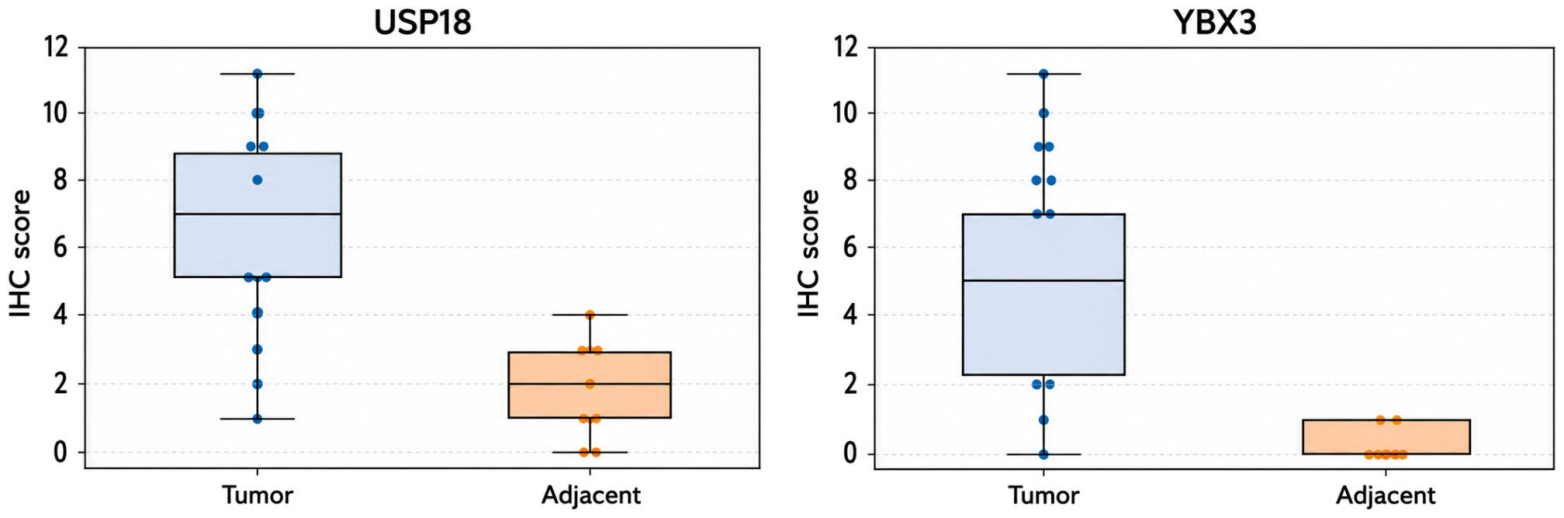
Tumor-versus-adjacent immunohistochemical expression of USP18 and YBX3. USP18 and YBX3 IHC scores were significantly higher in tumor tissue than in paired adjacent renal tissue.

Both biomarkers showed higher expression in tumor tissue than in paired adjacent renal tissue. For USP18, the mean tumor IHC score was 6.90 ± 2.16 compared with 1.81 ± 1.02 in adjacent tissue, yielding a mean paired difference of 5.09. This difference was significant by paired t-test (t = 22.242, p < 0.001) and Wilcoxon signed-rank testing (Z = -8.562, p < 0.001). For YBX3, the mean tumor score was 5.09 ± 2.88 compared with 0.27 ± 0.44 in adjacent tissue, with a mean paired difference of 4.83. This difference was also significant by paired t-test (t = 15.991, p < 0.001) and Wilcoxon signed-rank testing (Z = -8.384, p < 0.001). These paired results support tumor-associated overexpression of USP18 and YBX3 (**Table 2**; **Figure 1**). Because the comparator consisted of patient-matched adjacent tissue rather than samples from independent non-cancerous individuals, these findings demonstrate within-patient tumor-associated expression differences but do not establish population-level differences between ccRCC and healthy kidneys.

**Table 2:** Paired comparison of USP18 and YBX3 immunohistochemical scores between ccRCC tissue and patient-matched adjacent non-neoplastic renal tissue (N = 98 matched pairs).

Biomarker	Tumor mean ± SD	Adjacent mean ± SD	Mean paired difference	Paired t-test	Wilcoxon signed-rank test
USP18	6.90 ± 2.16	1.81 ± 1.02	5.09	t = 22.242, p < 0.001	Z = -8.562, p < 0.001
YBX3	5.09 ± 2.88	0.27 ± 0.44	4.83	t = 15.991, p < 0.001	Z = -8.384, p < 0.001

The IHC score ranged from 0 to 12 and was calculated as staining intensity (0-3) multiplied by the proportion score (0-4). Mean paired difference was calculated as tumor score minus adjacent-tissue score. Both the paired t-test and Wilcoxon signed-rank test are reported because the score distributions were not assumed to be normally distributed. Tests were two-sided. The adjacent tissue is a matched internal comparator and not an independent healthy control group. IHC, immunohistochemistry; SD, standard deviation.

### 3.3 Distribution of USP18 and YBX3 expression and clinicopathological associations

**Figure 2:**
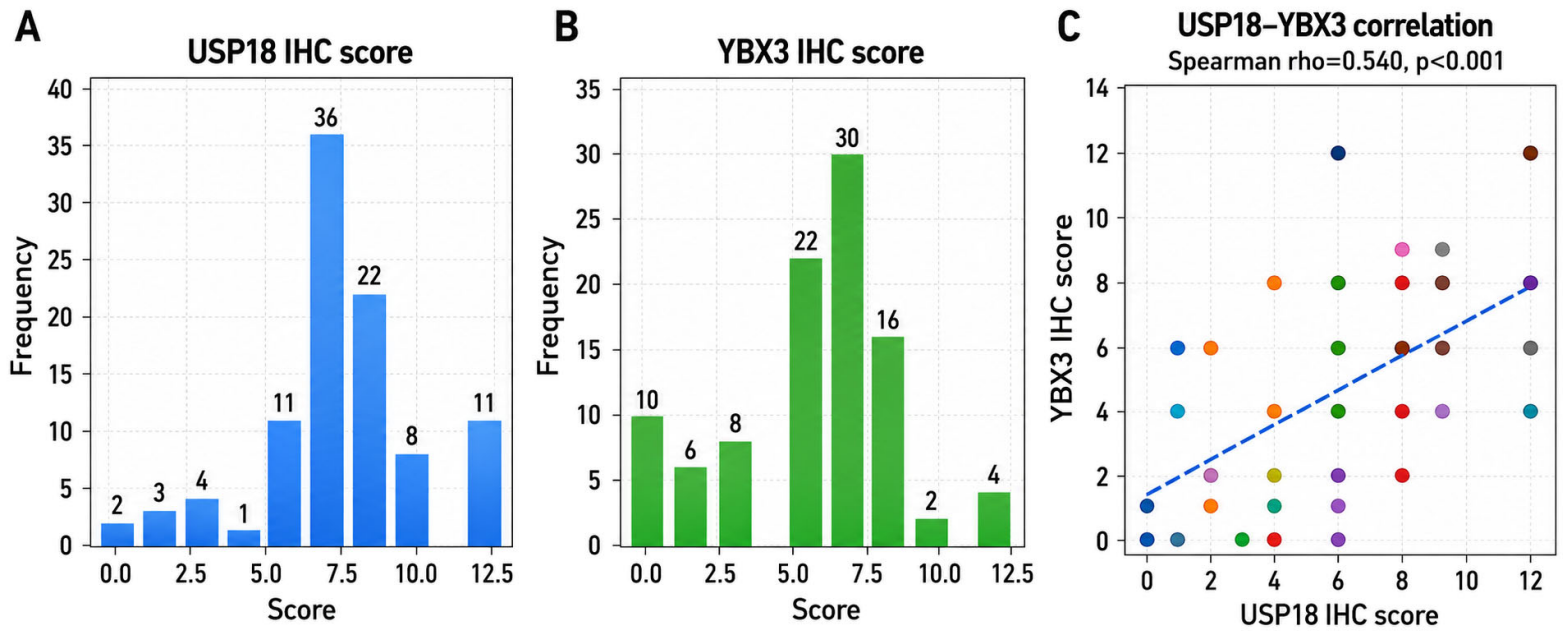
Distribution and correlation of USP18 and YBX3 IHC scores. **(A)** USP18 IHC score distribution. **(B)** YBX3 IHC score distribution. Numerical frequencies are shown above each bar to permit direct reading of the plotted data. **(C)** Scatter plot showing a significant positive correlation between continuous USP18 and YBX3 scores. Point colors are used only to improve visual separation of overlapping observations and do not encode additional clinical groups. The dashed line represents the linear trend. The correlation demonstrates co-expression and does not establish statistical synergy. IHC, immunohistochemistry.

Using the cohort-median IHC threshold of ≥6, USP18-high expression was observed in 77 cases (78.6%), whereas 21 cases (21.4%) were classified as USP18-low. YBX3-high expression was present in 52 cases (53.1%), and 46 cases (46.9%) were classified as YBX3-low. The median score for both biomarkers was 6.00, while the mean scores were 6.67 ± 2.83 for USP18 and 4.94 ± 2.95 for YBX3 (**Table 3**; **Figures 2A, B**). Numerical frequencies are displayed above the histogram bars in the revised **Figure 2** to permit direct reading of the plotted distributions.

**Table 3:** Distribution and exploratory categorical classification of USP18 and YBX3 immunohistochemical expression in ccRCC tumor tissue (N = 98).

Biomarker	Mean ± SD	Median (IQR)	Range	Low expression, n (%)	High expression, n (%)
USP18	6.67 ± 2.83	6.00 (6.00-8.00)	0-12	21 (21.4)	77 (78.6)
YBX3	4.94 ± 2.95	6.00 (4.00-6.00)	0-12	46 (46.9)	52 (53.1)

IHC scores ranged from 0 to 12. Low and high categories were defined using the cohort-median threshold: low expression, score <6; high expression, score ≥6. This threshold was used for descriptive and Kaplan-Meier presentation only, was not derived in an external cohort, and is not a clinically validated cutoff. Continuous scores were preferred for regression modeling. IHC, immunohistochemistry; SD, standard deviation; IQR, interquartile range.

Categorical USP18-high status was not significantly associated with sex, age group, surgical approach, tumor size group, vascular invasion, lymph-node/distant-organ metastasis, stage group, grade group, metastasis/recurrence, capsular invasion, necrosis, or death. YBX3-high status was likewise not significantly associated with most categorical clinicopathological variables; metastasis/recurrence showed only a borderline association (Pearson p = 0.057; Fisher exact two-sided p = 0.084). These predominantly negative associations do not support USP18 as a standalone clinicopathological marker in this cohort. Because the cohort was modest, however, absence of statistical significance cannot exclude smaller associations (**Table 4**).

**Table 4:** Associations between high USP18 or YBX3 expression and selected clinicopathological features in the 98-patient ccRCC cohort.

Variable	USP18 high: event/total (%)	p value	YBX3 high: event/total (%)	p value
Male sex	49/77 (63.6)	0.884	34/52 (65.4)	0.644
Age >55 years	42/77 (54.5)	0.832	29/52 (55.8)	0.888
Radical surgery	48/77 (62.3)	0.971	35/52 (67.3)	0.272
Tumor size >7 cm	11/77 (14.3)	1.000	9/52 (17.3)	0.363
Vascular invasion present	18/77 (23.4)	1.000	13/52 (25.0)	0.704
Lymph-node/distant metastasis present	9/77 (11.7)	0.084	8/52 (15.4)	0.982
Stage III-IV	6/77 (7.8)	0.398	3/52 (5.8)	0.298
Grade 3-4	36/77 (46.8)	0.944	26/52 (50.0)	0.519
Metastasis/recurrence	17/77 (22.1)	1.000	15/52 (28.8)	0.057
Capsular invasion	56/77 (72.7)	0.906	36/52 (69.2)	0.448
Necrosis	9/77 (11.7)	0.716	8/52 (15.4)	0.313
Death	10/77 (13.0)	1.000	7/52 (13.5)	0.696

High expression was defined as an IHC score ≥6. For each biomarker, the numerator is the number of biomarker-high tumors with the listed feature and the denominator is the total number of biomarker-high tumors; percentages therefore describe feature frequency within the biomarker-high subgroup. Associations comparing high and low expression groups were assessed using Pearson chi-square tests or two-sided Fisher exact tests when expected cell counts were small. The predominantly non-significant USP18 results do not support USP18 as a standalone clinicopathological marker. IHC, immunohistochemistry; ccRCC, clear cell renal cell carcinoma.

### 3.4 USP18-YBX3 relationship

USP18 and YBX3 were associated at both categorical and continuous levels. Among USP18-low tumors, 17/21 (81.0%) were YBX3-low and 4/21 (19.0%) were YBX3-high, whereas among USP18-high tumors, 29/77 (37.7%) were YBX3-low and 48/77 (62.3%) were YBX3-high. The categorical association was significant (chi-square = 12.415, p < 0.001; Phi = 0.356). Continuous-score analysis showed a moderate positive correlation (Spearman rho = 0.540, p < 0.001; Pearson r = 0.516, p < 0.001) (**Table 5**; **Figure 2C**). This relationship represents tissue-level co-expression and does not, by itself, demonstrate biological or statistical interaction.

**Table 5:** Categorical association and continuous-score correlation between USP18 and YBX3 expression in ccRCC tumor tissue (N = 98).

Analysis domain	Comparison	Statistic	p value
Grouped high/low association	USP18 high vs YBX3 high	Chi-square = 12.415; Phi = 0.356	<0.001
Continuous-score correlation	USP18 score vs YBX3 score	Spearman rho = 0.540	<0.001
Continuous-score correlation	USP18 score vs YBX3 score	Pearson r = 0.516	<0.001

Categorical high/low status used the cohort-median IHC threshold of ≥6. The chi-square statistic evaluates association between categorical expression groups, and Phi summarizes its effect size. Spearman rho was the primary rank-based measure for continuous IHC scores; Pearson r is provided as a complementary linear correlation estimate. All p-values are two-sided. These analyses demonstrate co-expression but do not establish biological or statistical interaction or synergy. IHC, immunohistochemistry.

### 3.5 Survival analysis

**Figure 3:**
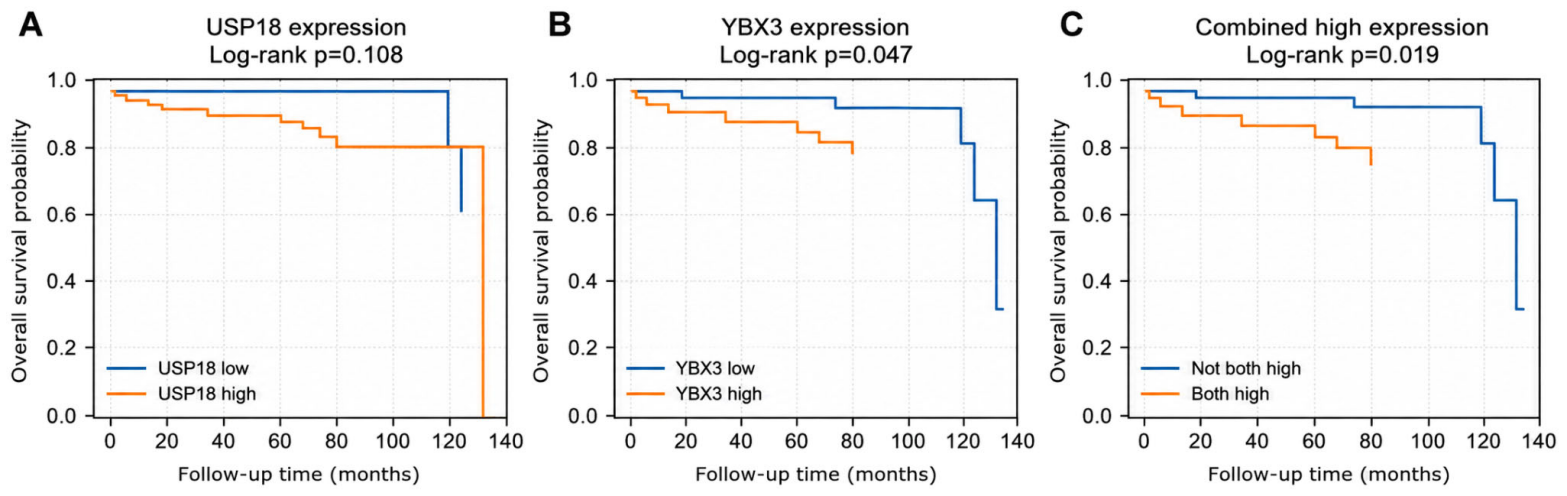
Kaplan-Meier overall survival curves according to biomarker expression. **(A)** USP18 high versus low expression. **(B)** YBX3 high versus low expression. **(C)** Combined high expression of both biomarkers versus not both high. The cutoff was cohort-median based, and the combined analysis is exploratory.

Kaplan-Meier analysis showed numerically shorter overall survival among USP18-high tumors, but the categorical comparison was not statistically significant (log-rank chi-square = 2.580, p = 0.108). In contrast, YBX3-high expression was associated with inferior overall survival (log-rank chi-square = 3.938, p = 0.047). Combined high expression of both biomarkers showed the clearest exploratory separation (log-rank chi-square = 5.538, p = 0.019), while the three-level biomarker-count model showed a near-significant trend (p = 0.054). Given the data-driven median cutoff, small number of events, and absence of a formal interaction test, the combined result should be interpreted as hypothesis-generating rather than evidence of synergy (**Table 6**; **Figure 3**).

**Table 6:** Kaplan-Meier overall-survival summary according to USP18, YBX3, and combined biomarker expression.

Group	N	Events	Censored	Mean follow-up/survival time, months	Log-rank chi-square	p value
USP18 low	21	2	19	105.38 ± 18.93	2.580	0.108
USP18 high	77	10	67	62.87 ± 33.12		
YBX3 low	46	5	41	88.11 ± 28.83	3.938	0.047
YBX3 high	52	7	45	57.71 ± 34.41		
Not both high	50	5	45	89.22 ± 28.25	5.538	0.019
Both high	48	7	41	54.02 ± 32.91		

Overall survival was measured from surgery to death from any cause or last contact. Events are deaths; patients alive or lost to follow-up were censored at last contact. High expression was defined as IHC score ≥6. “Not both high” includes all patients except those with high expression of both biomarkers. Mean follow-up/survival time is shown as mean ± standard deviation. Between-group survival distributions were compared using two-sided log-rank tests. Results are exploratory because the cutoff was cohort-derived and only 12 deaths occurred. IHC, immunohistochemistry.

### 3.6 Univariable Cox regression and exploratory multivariable sensitivity analysis

**Figure 4:**
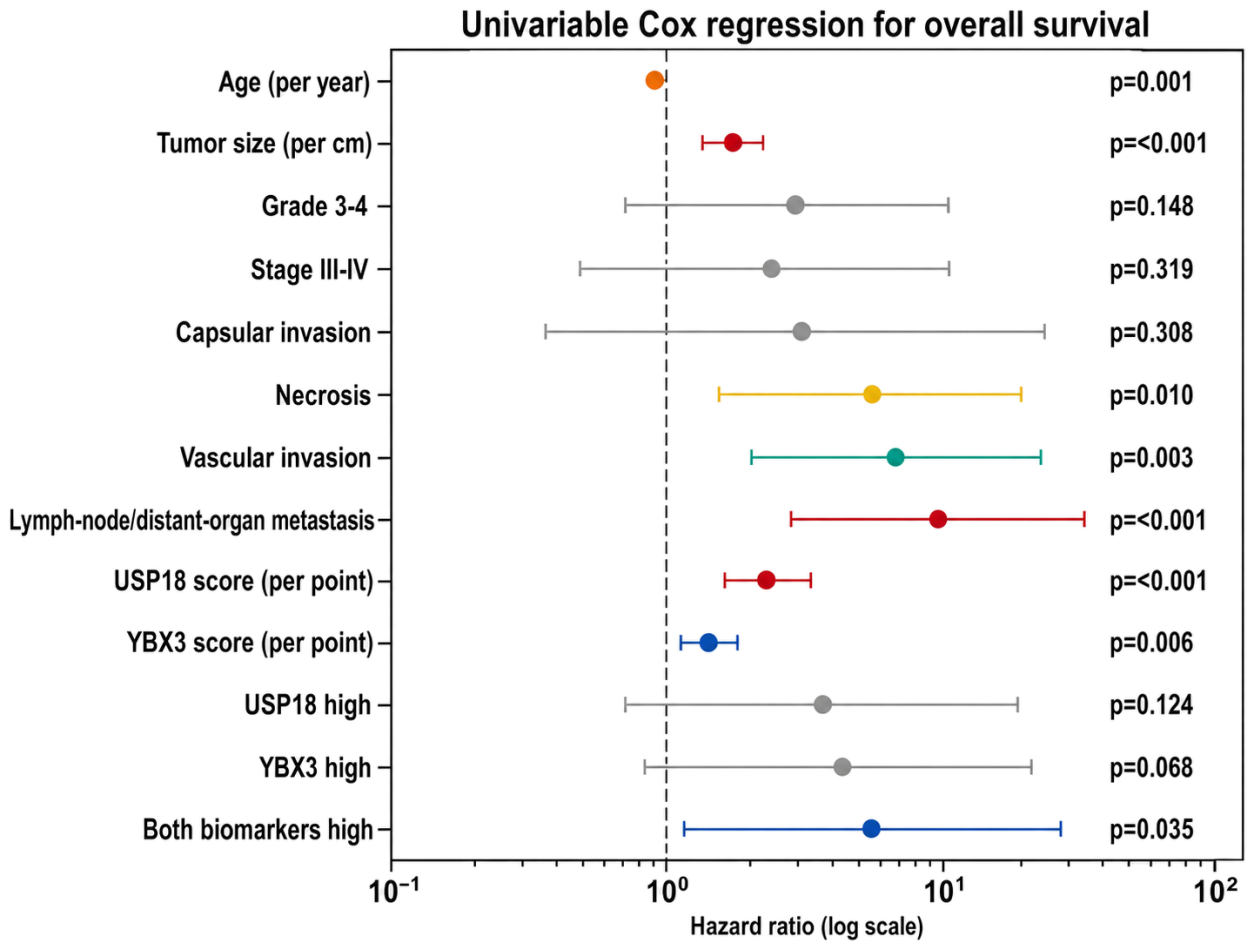
Univariable Cox regression forest plot for overall survival. Hazard ratios are shown on a logarithmic scale, with horizontal lines representing 95% confidence intervals. Gray denotes non-significant estimates (p ≥ 0.05), whereas colored points denote statistically significant estimates (p < 0.05). Distinct colors among significant estimates are used only for visual differentiation and do not represent additional statistical categories. Statistical interpretation is based on the numerical estimates and confidence intervals reported in **Table 7**.

In univariable Cox regression, age, tumor size, necrosis, vascular invasion, lymph-node/distant-organ metastasis, continuous USP18 score, and continuous YBX3 score were associated with overall survival. Each one-point increase in USP18 score was associated with increased mortality risk (HR 2.28, 95% CI 1.62-3.22, p < 0.001), and each one-point increase in YBX3 score was also associated with increased mortality risk (HR 1.36, 95% CI 1.09-1.69, p = 0.006). However, categorical USP18-high status was not significant (HR 3.59, 95% CI 0.71-18.28, p = 0.124), and categorical YBX3-high status was borderline (HR 4.34, 95% CI 0.89-21.01, p = 0.068). Combined high expression was associated with mortality in univariable analysis (HR 5.46, 95% CI 1.12-26.57, p = 0.035) (**Table 7**; **Figure 4**). The discordance between continuous and dichotomized USP18 results underscores the sensitivity of inference to cutoff selection.

**Table 7:** Univariable Cox proportional-hazards regression analyses for overall survival in the ccRCC cohort (N = 98; 12 deaths).

Variable	Hazard ratio	95% CI	p value
Age (per year)	0.92	0.88-0.97	0.001
Tumor size (per cm)	1.58	1.30-1.91	<0.001
Grade 3-4	2.69	0.70-10.29	0.148
Stage III-IV	2.20	0.47-10.40	0.319
Capsular invasion	2.94	0.37-23.30	0.308
Necrosis	5.29	1.48-18.86	0.010
Vascular invasion	6.52	1.91-22.30	0.003
Lymph-node/distant-organ metastasis	9.33	2.72-32.03	<0.001
USP18 score (per point)	2.28	1.62-3.22	<0.001
YBX3 score (per point)	1.36	1.09-1.69	0.006
USP18 high	3.59	0.71-18.28	0.124
YBX3 high	4.34	0.89-21.01	0.068
Both biomarkers high	5.46	1.12-26.57	0.035

Hazard ratios for age, tumor size, and continuous biomarker scores represent the change associated with a one-unit increase. Binary-variable hazard ratios compare the listed category with its complementary reference category. Models were fitted separately for each variable and are not mutually adjusted. Continuous and dichotomized biomarker findings should be interpreted together; wide confidence intervals for categorical biomarkers and the non-significant categorical USP18 estimate limit inference about a clinically usable threshold. HR, hazard ratio; CI, confidence interval; ccRCC, clear cell renal cell carcinoma.

Because only 12 deaths were observed, a stable multivariable model containing the full set of clinical variables and biomarkers could not be supported. The previously fitted multivariable models have therefore been moved to **Supplementary Table 1** and are presented only as exploratory sensitivity analyses. Their large hazard ratios and very wide confidence intervals are consistent with model instability and do not establish independent prognostic effects.

### 3.7 Exploratory clinical prediction modeling and ROC analysis

**Figure 5:**
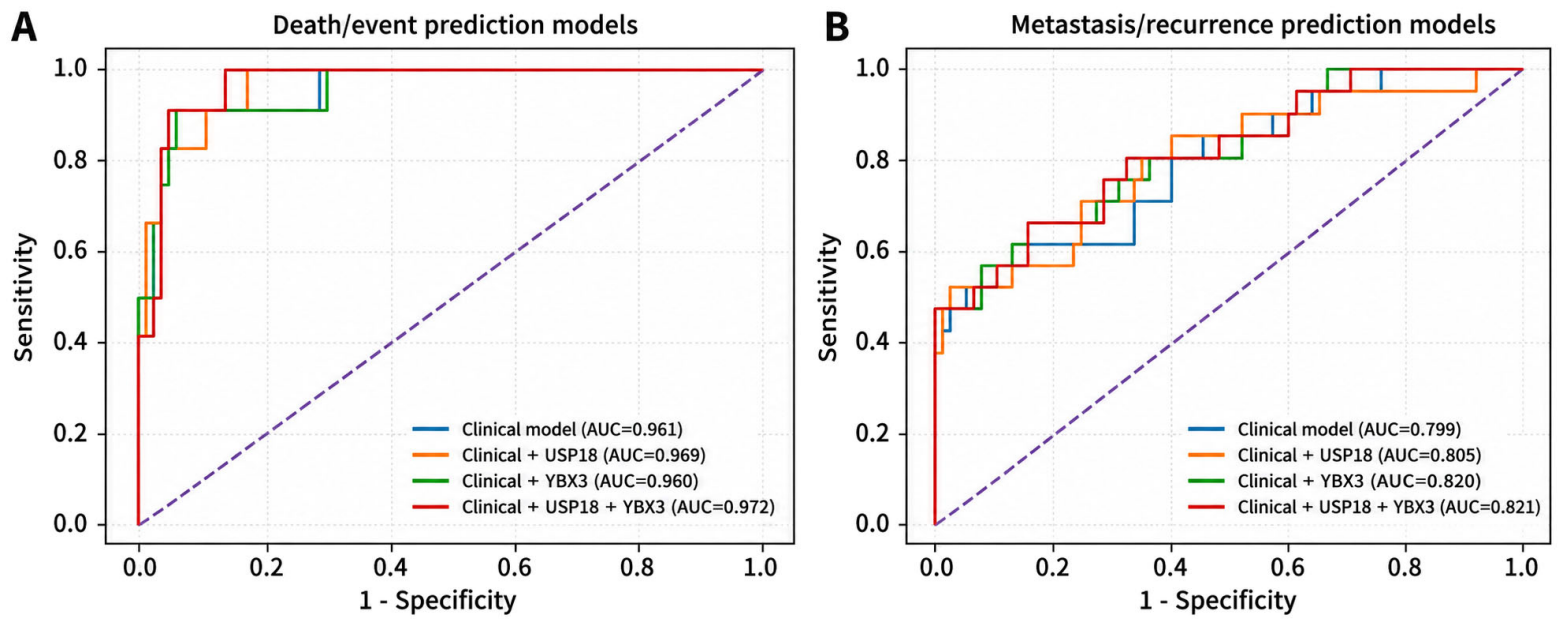
ROC curves for clinical and biomarker-added prediction models. **(A)** Death/event prediction. **(B)** Metastasis/recurrence prediction. The horizontal axis displays specificity in decreasing order from 1.0 to 0.0 from left to right, which is mathematically equivalent to the conventional increasing 1-specificity scale. Curves represent apparent in-sample performance and were not externally validated.

For death/event prediction, the clinical model alone showed an apparent AUC of 0.961. Adding USP18 yielded an AUC of 0.969, adding YBX3 yielded an AUC of 0.960, and adding both biomarkers yielded an AUC of 0.972. For metastasis/recurrence prediction, corresponding AUCs were 0.799, 0.805, 0.820, and 0.821. The absolute increases over the clinical model were small, and all models were developed and evaluated in the same retrospective cohort. These values therefore describe apparent in-sample discrimination and should not be interpreted as validated incremental predictive value or clinical utility (**Table 8**; **Figure 5**).

**Table 8:** Apparent discrimination of clinicopathological and biomarker-added logistic prediction models for death/event and metastasis/recurrence.

Outcome	Model	N	Events	AUC	95% CI	p value
Death/event	Clinical model	98	12	0.961	0.914-1.000	<0.001
Death/event	Clinical + USP18	98	12	0.969	0.934-1.000	<0.001
Death/event	Clinical + YBX3	98	12	0.960	0.911-1.000	<0.001
Death/event	Clinical + USP18 + YBX3	98	12	0.972	0.941-1.000	<0.001
Metastasis/recurrence	Clinical model	98	21	0.799	0.678-0.920	<0.001
Metastasis/recurrence	Clinical + USP18	98	21	0.805	0.686-0.924	<0.001
Metastasis/recurrence	Clinical + YBX3	98	21	0.820	0.704-0.936	<0.001
Metastasis/recurrence	Clinical + USP18 + YBX3	98	21	0.821	0.705-0.936	<0.001

All models used the same retrospective cohort (N = 98), with 12 death events and 21 metastasis/recurrence events. The clinical model included age, tumor size, grade group, stage group, capsular invasion, necrosis, vascular invasion, and lymph-node/distant-organ metastasis where appropriate. Biomarker models added continuous USP18 score, continuous YBX3 score, or both. AUCs and 95% confidence intervals are apparent in-sample estimates because model development and evaluation used the same dataset. Reported p-values test discrimination against the null AUC of 0.50 and do not test differences between models. Small AUC changes were not externally validated and do not establish incremental clinical utility. AUC, area under the receiver operating characteristic curve; CI, confidence interval.

### 3.8 Representative H&E and tumor-versus-adjacent IHC findings

**Figure 6:**
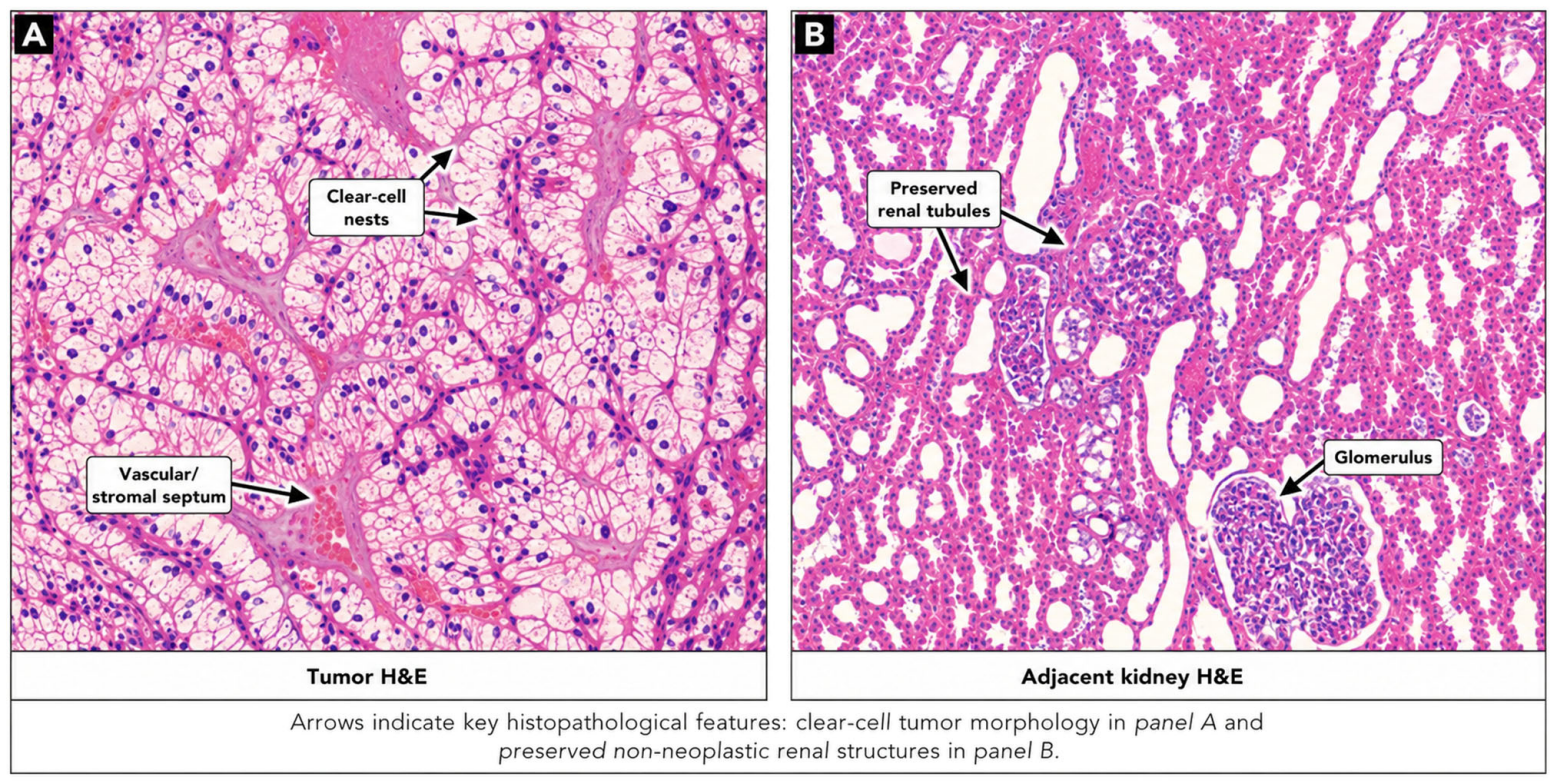
Representative H&E morphology of ccRCC tumor and patient-matched adjacent non-neoplastic kidney. **(A)** Tumor tissue showing clear-cell morphology and nested/alveolar tumor architecture; arrows indicate representative clear-cell tumor nests and a vascular/stromal septum. **(B)** Adjacent kidney tissue showing preserved non-neoplastic renal tubules and a representative glomerulus, indicated by arrows. The adjacent tissue provides a matched histological comparator but is not an independent healthy control. H&E, hematoxylin and eosin; ccRCC, clear cell renal cell carcinoma.

**Figure 7:**
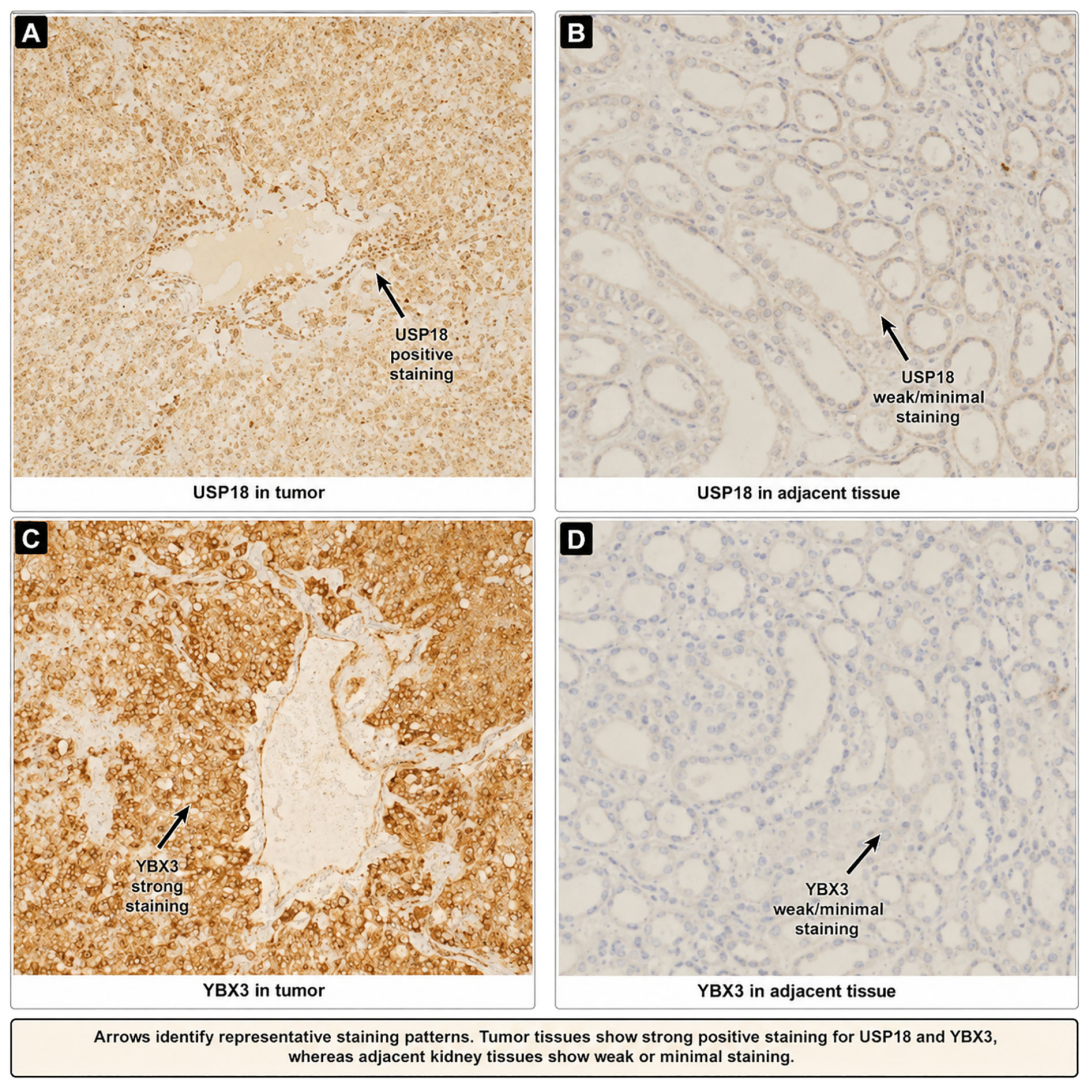
Representative tumor-versus-adjacent immunohistochemical staining for USP18 and YBX3. **(A)** USP18 staining in ccRCC tumor tissue; the arrow indicates representative positively stained tumor cells. **(B)** USP18 staining in paired adjacent non-neoplastic renal tissue; the arrow indicates an area of weak or minimal staining. **(C)** YBX3 staining in ccRCC tumor tissue; the arrow indicates representative strongly positive tumor cells. **(D)** YBX3 staining in paired adjacent renal tissue; the arrow indicates weak or minimal staining. Tumor and adjacent sections were processed in parallel. IHC, immunohistochemistry; ccRCC, clear cell renal cell carcinoma.

Representative hematoxylin and eosin images confirmed the paired tissue context used for tumor-versus-adjacent comparison. Tumor sections showed morphology consistent with ccRCC, including clear cytoplasm and nested/alveolar architecture, whereas adjacent kidney tissue retained non-neoplastic renal tubular and glomerular architecture. Representative IHC images showed stronger tumor staining for USP18 and YBX3 than adjacent kidney, supporting the quantitative paired findings (**Figures 6**, **7**).

## 4 Discussion

This study evaluated USP18 and YBX3 expression in ccRCC by integrating paired tumor-adjacent IHC, clinicopathological correlation, survival analysis, Cox regression, and exploratory ROC modeling. Both markers were overexpressed in tumor tissue and showed a moderate tissue-level correlation. The clinical evidence was stronger for YBX3 than for categorical USP18 expression, while the combined expression pattern was exploratory and should not be interpreted as proof of synergy or independent prognostic utility.

The paired tumor-adjacent comparison places the biomarker findings within routine histopathological material. USP18 and YBX3 expression was substantially higher in tumor than in adjacent non-neoplastic kidney, indicating tumor-associated upregulation. IHC is compatible with formalin-fixed, paraffin-embedded tissue and can therefore support retrospective validation and potential incorporation into pathology workflows if reproducibility and clinical validity are established. This integrative approach is consistent with broader ccRCC biomarker research that evaluates molecular markers alongside established clinicopathological variables (24).

A biologically informative observation was the positive association between USP18 and YBX3. YBX3-high expression was more frequent among USP18-high tumors, and the continuous IHC scores were moderately correlated. This clinical co-expression pattern is consistent with experimental findings that USP18 can regulate the ubiquitination and stability of YBX3 (25). Nevertheless, correlation does not establish that the combined effect exceeds the product of the individual effects. The present study therefore supports tissue-level co-expression, not statistical interaction or mechanistic causality.

The findings are also consistent with the broader involvement of ubiquitin-proteasome deregulation in renal cancer. Aberrant ubiquitination and deubiquitination influence HIF signaling, PI3K/AKT/mTOR signaling, ferroptosis, p53 regulation, immune escape, and therapeutic resistance in RCC (26). Deubiquitinating enzymes regulate proliferative signaling, cell-death resistance, metabolic reprogramming, phenotypic plasticity, and immune modulation (27). Within this context, USP18 may participate in a regulatory network that affects downstream effectors such as YBX3, but the present clinical study cannot establish the direction or mechanism of that relationship.

The survival findings require a distinction between continuous and dichotomized analyses. Categorical USP18-high status was not significantly associated with overall survival, whereas the continuous USP18 score was associated with mortality in univariable Cox analysis. This discordance weakens the argument for a clinically usable USP18 cutoff and suggests that the apparent association may depend on analytical scale. YBX3-high expression showed a statistically significant log-rank comparison, although the small number of deaths produced imprecise categorical hazard ratios. Accordingly, the current data do not support USP18 as a validated standalone prognostic marker.

Combined high expression showed the clearest Kaplan-Meier separation, but this finding must be interpreted conservatively. Only seven deaths occurred in the both-high group and five in the comparison group, the cutoff was based on the cohort median, and no adequately powered interaction model could be fitted. The combined pattern may identify a biologically coherent subgroup, but it does not demonstrate that the joint effect of USP18 and YBX3 exceeds a multiplicative relationship. Confirmation requires centered continuous-score interaction analysis in a substantially larger dataset.

The Cox regression findings further illustrate the risk of overfitting. With only 12 deaths, models containing several clinicopathological variables and one or two biomarkers have an unfavorable events-to-parameter ratio. Such models can fit random features of the derivation cohort, generate unstable coefficients, and yield exaggerated hazard ratios with wide confidence intervals. We therefore moved the expanded multivariable analyses to the **Supplementary Material** and based the main interpretation on univariable estimates, confidence-interval precision, and consistency across analyses. The multivariable results should be regarded as sensitivity analyses rather than evidence of independent prognostic value.

The ROC models provide only an exploratory translational perspective. The clinical model already showed very high apparent discrimination for death/event prediction, and adding biomarkers changed the AUC by only -0.001 to 0.011. For metastasis/recurrence, increases ranged from 0.006 to 0.022. These differences were not validated or tested in an independent cohort, and high in-sample AUCs can result from overfitting when the number of events is small relative to the number of predictors. Consequently, the results do not establish incremental clinical utility. Recent development and validation studies of multigene prognostic signatures in localized and metastatic ccRCC likewise emphasize that independent validation is essential before a biomarker model can be considered clinically actionable (28, 29).

### 4.1 Limitations of the study

Several limitations materially affect interpretation. The retrospective single-center design may introduce selection bias and limits generalizability. The cohort included only 98 patients, most with stage I-II disease, and only 12 deaths. This limited statistical power to detect small or moderate associations, increased the probability of type II error, widened confidence intervals, and constrained reliable adjustment for confounding. The non-significant categorical USP18 result may therefore reflect either a genuinely weak or absent effect or insufficient power; the present data cannot distinguish these possibilities. At the same time, non-significance cannot be used to support prognostic utility, and the available evidence does not establish USP18 as an independent marker. In addition, an independent non-cancer control group was not available. Although paired adjacent tissue reduces interpatient variability and controls partly for fixation and processing conditions, tissue adjacent to a tumor may contain field effects, age-related change, inflammation, ischemic alteration, or underlying renal disease and should not be assumed to be equivalent to kidney tissue from cancer-free individuals.

The small event count also made subgroup estimates, interaction testing, and multivariable models unstable. Strong correlation between USP18 and YBX3 further reduced the precision with which their independent contributions could be separated. Semi-quantitative IHC scoring may be affected by intratumoral heterogeneity, staining variability, and observer differences. Dichotomizing scores at the cohort median reduced information and created a threshold that has not been externally validated. The available sample was insufficient for reliable split-sample validation, and the clinical models lacked calibration assessment, decision-curve analysis, and external validation. Reproducibility is improved by verification of the Sigma-Aldrich primary-antibody identities, including the YBX3 catalog/SKU and lot number. However, the anti-USP18 lot number and product-specific catalog and lot identifiers, working dilutions, and exact operating conditions for the ancillary Sigma-Aldrich IHC and H&E reagents were not retained in the retrospective archive.

Future studies should use larger multicenter cohorts with longer follow-up, more advanced-stage cases, and substantially more outcome events. A prespecified validation protocol should retain biomarker scores continuously, define any categorical threshold before outcome analysis, assess interobserver reproducibility, and use penalization or other approaches appropriate to the number of events. Internal validation, calibration, decision-curve analysis, and independent external validation will be required before the USP18/YBX3 pattern can be considered for clinical risk stratification. Such studies should also include an independent non-cancer comparison group, such as histologically normal kidney tissue from appropriately consented non-malignant nephrectomy specimens or other ethically sourced control tissue, matched where feasible for age, sex, renal function, and pre-analytical processing. Complete reagent identifiers, antibody dilutions, staining-platform settings, and quality-control procedures should be prospectively recorded and reported.

## 5 Conclusion

USP18 and YBX3 are overexpressed and co-expressed in ccRCC tissue. YBX3 showed the stronger individual survival signal, whereas categorical USP18 expression was not associated with most clinicopathological variables and was not significant in univariable survival analysis. The continuous-score and combined-expression findings are preliminary and do not establish a clinically validated cutoff, statistical synergy, or independent prognostic utility. Larger externally validated cohorts with prespecified scoring procedures and adequately powered interaction analyses are required before either marker or their combination can be considered for clinical risk stratification.

## Data Availability

The raw data supporting the conclusions of this article will be made available by the authors, without undue reservation.
