# Supplementary material for: Clinicopathological significance and exploratory prognostic modeling of USP18 and YBX3 expression in clear cell renal cell carcinoma: a retrospective Chinese cohort study
Source: Front Oncol. 2026 Aug 4;16:1897469. doi: 10.3389/fonc.2026.1897469 (PMC13481323; doi:10.3389/fonc.2026.1897469)
Supplement: Supplementary file 1 [file Table1.docx]

***Supplementary Material***

**Clinicopathological Significance and Exploratory Prognostic Modeling of USP18 and YBX3 Expression in Clear Cell Renal Cell Carcinoma: A Retrospective Chinese Cohort Study**

# 1 **Supplementary Data**

The supplementary data provide the expanded multivariable Cox proportional-hazards analyses that were removed from the main manuscript to reduce the risk of overfitting and to maintain a conservative interpretation of the prognostic findings. These models are retained solely as exploratory sensitivity analyses and should be considered together with the univariable results reported in the main manuscript.

Only 12 deaths occurred in the 98-patient cohort. Consequently, the number of fitted parameters was high relative to the available outcome information, and the resulting estimates are vulnerable to overfitting, sparse-data bias, coefficient instability, and wide confidence intervals. The models therefore do not establish independent prognostic validity for USP18, YBX3, or their combined expression.

A USP18×YBX3 interaction term was considered but was not fitted because the small number of deaths and the moderate correlation between the continuous IHC scores would make an interaction estimate particularly unstable and underpowered. The study therefore reports tissue-level co-expression and exploratory combined-expression findings without claiming statistical synergy.

# 2 **Supplementary Figures and Tables**

## 2.1 **Supplementary Tables**

**Supplementary Table S1.** Expanded exploratory multivariable Cox proportional-hazards models for overall survival in the ccRCC cohort (N = 98; 12 deaths).

| **Model** | **Variable** | **Hazard ratio** | **95% CI** | **p value** |
| --- | --- | --- | --- | --- |
| Clinical model | Age (per year) | 0.93 | 0.86-1.00 | 0.055 |
| Clinical model | Tumor size (per cm) | 1.75 | 1.32-2.32 | <0.001 |
| Clinical model | Grade 3-4 | 2.25 | 0.51-10.04 | 0.286 |
| Clinical model | Stage III-IV | 0.33 | 0.04-2.85 | 0.314 |
| Clinical model | Capsular invasion | 23.25 | 1.29-420.03 | 0.033 |
| Clinical model | Necrosis | 2.25 | 0.40-12.58 | 0.355 |
| Clinical + USP18 | Tumor size (per cm) | 1.45 | 1.07-1.97 | 0.018 |
| Clinical + USP18 | Necrosis | 13.73 | 1.60-118.16 | 0.017 |
| Clinical + USP18 | USP18 score | 2.85 | 1.53-5.31 | <0.001 |
| Clinical + YBX3 | Tumor size (per cm) | 1.78 | 1.32-2.40 | <0.001 |
| Clinical + YBX3 | YBX3 score | 1.46 | 1.09-1.95 | 0.011 |
| Clinical + both biomarkers | Tumor size (per cm) | 1.43 | 1.04-1.97 | 0.026 |
| Clinical + both biomarkers | Necrosis | 13.01 | 1.42-119.13 | 0.023 |
| Clinical + both biomarkers | USP18 score | 2.61 | 1.33-5.12 | 0.005 |
| Clinical + both biomarkers | YBX3 score | 1.15 | 0.69-1.91 | 0.601 |

*Note.* Models were fitted as exploratory sensitivity analyses because only 12 deaths were available and the number of fitted parameters was high relative to the event count. Hazard ratios and 95% confidence intervals may therefore be unstable and should not be used to claim independent biomarker validation. The clinical model included age, tumor size, grade group, stage group, capsular invasion, and necrosis; biomarker-added models included continuous USP18 and/or YBX3 IHC scores as shown. The reported rows reproduce the retained model output for transparency. HR, hazard ratio; CI, confidence interval; IHC, immunohistochemistry; ccRCC, clear cell renal cell carcinoma.
